# An Investigation of the Relationship between Personality, Cognitive Ability, and Work Engagement in Intellectually Gifted Individuals

**DOI:** 10.3390/jintelligence10040100

**Published:** 2022-11-10

**Authors:** Lindsey Macke, Flor de León, Tobias Hermansson, Petri Kajonius

**Affiliations:** 1 Department of Psychology, Lund University, 221 00 Lund, Sweden; 2 Mensa, Mensa Sweden, 120 31 Stockholm, Sweden

**Keywords:** Mensa, personality traits, Big Five, Mini-IPIP, honesty-humility, intelligence, work engagement

## Abstract

Do personality traits in highly intelligent individuals relate to their work engagement? Seemingly little is known about the relationship between personality and work engagement for gifted individuals. In what may be the first study to do so, a Swedish Mensa sample (*n* = 353) was explored with a two-part aim: to assess psychometric personality properties and to investigate the relationship between personality traits (Mini-IPIP6) and work engagement (UWES-9). The results of the Mensa members and the Mturk sample (1.4 SD lower in cognitive ability based on ICAR-16) were compared using a confirmatory factor analysis (CFA) and a regression. The findings indicated that the Mensa sample had higher openness (*d* = .50) and honesty-humility (*d* = .65) and that personality traits were similarly related to work engagement in both groups, with the exception that neither openness nor honesty-humility were related to work engagement in the Mensa sample. The characteristics of intellectually gifted individuals are further discussed.

## 1. Introduction

In the intellectual giftedness literature, there is debate regarding the social characteristics of high-IQ individuals. Some argue that gifted persons are more prone to affective disorders and interpersonal maladjustments due to inadequate support (Karpinski et al. 2018; Matta et al. 2019). Others argue that, compared to average populations, gifted individuals tend to have personality traits conducive for social engagements (Ogurlu 2021). A three-level meta-analysis that explored the Big Five personality dimensions and giftedness in 13 publications revealed that gifted people score significantly higher in the openness to experience dimension compared to an average control sample (Ogurlu and Özbey 2021). Such findings suggest that the intellectually gifted navigate the world in a unique way compared to the average population.

According to Rinn and Bishop (2015), the intelligence literature tends to focus more on the childhood implications of intellectual giftedness rather than future trajectories. For example, how do these individuals behave when their daily environment is no longer school but work? In convenience sampling, employee engagement has been found to be positively correlated with extraversion, conscientiousness, and openness to experience, while neuroticism has been found to be negatively correlated with work engagement (Karimi 2019). However, personality research such as this seldom takes cognitive ability into account. Consequently, little is known regarding how gifted adults manage roles where their intellectual capacity may be affected by cognitive challenges such as boredom (Salgado and Moscoso 2019). To better understand the work lives of gifted adults and explore how their personality traits may predict work engagement, we investigated the effects of personality on work engagement in a unique sample consisting of Mensa members. 

### 1.1. Gifted Individuals

Evidence for interpersonal propensities and emotional acuity in this population contradict the “mad genius” notion popularized during the Victorian era by suggesting gifted individuals have social-cognitive differences but not deficiencies (Stiles 2009). Cognitive ability, high-IQ, intellectual giftedness, and gifted, all refer to an individual with high general intelligence (g-factor). While intelligence can take on many meanings, general intelligence is historically defined as the ability to perform well on various cognitive tasks (DeYoung 2020). 

Studying the relationship between personality and work engagement in high-IQ individuals requires intelligence screening. Because of this, there has been increased interest in recruiting members from groups such as the Mensa Association (i.e., the high-IQ Society). The Mensa Society is an international organization of high-IQ members who have a tested IQ above 130 (Mensa International 2022). The use of this group as a proxy for intelligence studies was inspired by a researcher who discerned the difficulty in identifying high-IQ individuals in public samples (Fogel 1968). 

### 1.2. Mensa Members at Work

The present study concerns the personality traits of the intellectually gifted and how they relate to work engagement. According to Schaufeli and Bakker (2004), work engagement refers to how connected one feels toward their work and how competent one feels in managing the demands of their job. It is characterized by a strong identification with one’s work and is the supposed antonym of burnout. Work engagement is an effective way to measure the health of worker–employer relationships (Schaufeli and Bakker 2004). Nevertheless, work engagement research has mostly focused on average community samples, as opposed to high-IQ groups. While the literature on intelligence specifically relating to work engagement is scarce, there is some evidence for intelligence being amongst the most powerful predictors of work performance (Schmidt and Hunter 2004; Salgado and Moscoso 2019). When tasks are not sufficiently challenging, cognitive ability may also be a predictor of workplace attrition (Salgado and Moscoso 2019). The lack of literature on this topic may inhibit gifted individuals and their employers from exploring their full potential or result in preventable workplace dissatisfaction. Thus, it is essential to understand the relationship between personality and work engagement for those with high cognitive ability.

### 1.3. Personality and Work Engagement

Extensive empirical evidence in personality psychology suggests that the Big Five framework by Goldberg (1981) is a reliable model for assessing personality according to five domains: extraversion, neuroticism, agreeableness, conscientiousness, and openness (Costa and McCrae 1992). These five personality domains contain a number of lower-order facets, or traits, based on a pool of items (Kajonius and Johnson 2019). Over the years, new personality dimensions have been identified. Such is the case for the Big Six model of personality structure based on the HEXACO model (Ashton et al. 2007). This model is an alternative to the five-factor model and is believed to account for individual differences related to sincerity and modesty by including an additional dimension for honesty-humility (Oda and Matsumoto-Oda 2022). Notably, this dimension of honesty-humility seems to be particularly useful for predicting cheating and taking shortcuts (Kajonius 2014). 

The Mini-IPIP6 scale (Sibley et al. 2011) is a personality assessment instrument derived from the Mini-IPIP (Goldberg 1999), which is based on the Big Six model. While the original Mini-IPIP five-factor structure was developed and validated across multiple studies through exploratory and confirmatory factor analyses (Cooper et al. 2010), the Mini-IPIP6 was assessed through item response models (Sibley 2012). It provides reasonably distributed estimates for each of its six dimensions of personality, although little is known about its reliability and criterion validity in other samples. Personality models and instruments such as this have only begun to explore the connection between personality and work behavior.

A recent meta-analysis explored the relationship between personality and work engagement in community samples (Fukuzaki and Iwata 2022). Based on 36 published papers, the Big Five model could explain 30% of work engagement variance. Furthermore, conscientiousness demonstrated the strongest association (ρ = .41) with work engagement, followed by extraversion and openness to experience (.38), neuroticism (−.36), and agreeableness (.27). Similarly, in a study on university staff, the Big Five model could explain 40% of the variance in work engagement (Karimi 2019). 

### 1.4. Present Study

While intelligence is a topic of great research interest, there have been limited and contradicting findings regarding personality traits in gifted individuals, and there is a particular gap in the research related to workplaces and work engagement. By exploring data from Mensa members, this study endeavored to further understand this connection in a unique sample. 

Even though the structure from the Mini-IPIP6 scale was derived from its parent measure, the Mini-IPIP based on the Big Five, Smith et al. (2000) pointed out that researchers often fail to evaluate whether a new form has a comparable amount of reliability and validity. Thus, it is important to assess if an instrument can generalize across independent samples to allow continued progress. Therefore, the aims of the present study are as follows:

- Present and assess the psychometric properties of personality traits in gifted individuals.
- Present and assess whether personality traits differ in predicting work engagement between gifted individuals and a control sample.

## 2. Materials and Methods

### 2.1. Participants and Procedure

The data used in this study were originally collected for an unpublished investigation regarding Mensa members’ work–life balance. Researchers recruited participants by advertising their survey via the Swedish Mensa Association newsletter in 2018 (*n =* 359; estimated response rate 23% based on the number of readers). A link to a website survey with instructions on filling out a personality form and work-related questions was given. The instructions emphasized that data would be used for a future psychology research study, with the hope of enhancing the quality of input data. Additionally, a comparison sample of a similar size (*n* = 304) was collected via Amazon Mechanical Turk (MTurk), which is known to deliver high-quality data. The participants who signed up for the task had to have over 95% approval rates from previous Mturk data collections, which is a normative quality marker for data input on Mturk (Chmielewski and Kucker 2020). Similar instructions were provided for the control group; however, it was presented as a regular research data collection, and no mention of the Mensa comparison was given. The characteristics of the study variables in both samples are found in Table 1. Eighteen participants were excluded from further analysis due to sporadic responses. The Mturk sample was 65% men and had a total sample mean age of 32.3 (SD = 8.8). Similarly, the Mensa sample was 65% men and had a mean age of 39.0 (SD = 10.6). The educational levels (0–8) were the same in both samples (M = 6.3, SD = 1.2, bachelor’s degree). The sex differences in both samples were as expected (Kajonius and Johnson 2018), with significant differences in the personality traits of agreeableness, honesty-humility, and neuroticism (*d* = .5–.7) and no differences found in cognitive ability or work engagement. The final dataset included *N* = 639 participants (*n =* 353 Mensa members). 

### 2.2. Measurements

Based on the International Standard Classification of Education, demographic information regarding age, binary gender, and educational level (0–8, where 0 = no education and 8 = doctoral degree) was used (Schneider 2022). Work sector data (free-text qualitative answers) were only collected as part of the original collection (not in the Mturk comparison sample) and thus were not analyzed. Given the generally high English proficiency among Swedish people, especially those in a high-IQ society such as Mensa, the original English versions of all instruments were used.

### 2.3. Work Engagement

The Utrecht work engagement short scale (UWES-9) is a nine-item self-report questionnaire to assess work engagement. Participants respond on a 7-point Likert scale ranging from 0 = Never to 6 = Always. The scale provides a total score for self-perceived work engagement (Schaufeli and Bakker 2004). The scale has good internal consistency, with Cronbach’s alpha values equal to or exceeding the critical value of .70 (Nunnally and Bernstein 1994). The UWES-9 consists of a three-factor structure: vigor, dedication, and absorption. Vigor is defined as a high level of energy while working, dedication refers to the level of involvement in one’s work, and absorption refers to being fully engrossed in work (Schaufeli and Bakker 2004). 

### 2.4. Personality Traits

The Mini-IPIP6 scale (Sibley et al. 2011) is an instrument derived from the five-factor model of personality by Goldberg (1981). It is a 24-item self-report questionnaire that aims to measure six broad personality traits: extraversion, neuroticism, agreeableness, conscientiousness, openness to experience, and honesty-humility. Participants respond using a 7-point Likert scale ranging from 1 = Very inaccurate to 7 = Very accurate. This scale is based on the Big Six model, also known as HEXACO, which incorporated a sixth distinct dimension for honesty-humility (Ashton et al. 2007). The scale has a good test information function (TIF) in analyses using item response theory and good internal consistency, with most items’ alpha values exceeding the critical value of .70 (Sibley et al. 2011). The item numbers are the same as in the original Sibley et al. (2011) study.

### 2.5. Intelligence

The ICAR-16 (Condon and Revelle 2014; The ICAR Team 2014) is an intelligence test that consists of 16 items. ICAR is an international collaboration (https://icar-project.com/), that is intended to serve researchers with items to pick and choose from. For the present study, four items were chosen, one for each corresponding cognitive ability, plus four items for three-dimensional rotations. Such rotating cubes are reported to be among the more difficult tasks (Condon and Revelle 2014) and are therefore potentially suitable to differentiate intelligence, even among Mensa members. In total, our intelligence test consisted of eight items, which were half of the original ICAR-16 items. Seeing the internal consistency on these items in the present study (α = .78), this was arguably a suitable measurement of cognitive ability. The ICAR-16 has been used in Swedish settings before (Kajonius and Björkman 2020), reporting an alpha of .74. A correct answer was scored with one point, and only one answer per item could be correct. ICAR has exhibited good psychometric properties, and ICAR-16 correlates highly with Wechsler’s full Adult Intelligence Scale (WAIS) (*r* = .85) (Condon and Revelle 2014). 

### 2.6. Statistical Analysis

This was a cross-sectional study on high-IQ individuals who are part of the Mensa Society in Sweden. The dataset was screened, and all multivariate assumptions (i.e., normality, linearity, and multicollinearity) were met. An examination of the reliability of the Mini IPIP6 scale was prioritized to adequately assess and understand personality in future research on similarly unique samples. To our knowledge, this study is the first of its kind to assess the internal reliability of the six-factor structure from the Mini-IPIP6 scale through a confirmatory factor analysis (CFA) in a unique sample of gifted individuals. To evaluate the Mini-IPIP6 in a unique sample, the analyses were conducted on the Mensa member sample using the JASP computer software (JASP Team 2022) and Jamovi (Jamovi Project 2021).

The homogeneity of variance-covariance was assessed visually with residual plots, and no major violations of the assumption were spotted. The linearity and normality were checked through a hypothetical regression. For this analysis, 18 participants were excluded due to sporadic answering. All analyses were conducted with *n* = 353 Mensa members. Sampling adequacy was individually assessed using the Kaiser–Meyer–Olkin measure of sampling adequacy (MSA); all the items were >.5, as recommended by Hair et al. (2006). Bartlett’s test indicated correlation adequacy (χ^2^ (276) = 2755.58, *p* < .001), suggesting that there may be a statistically significant interrelationship between variables in the dataset. The KMO test indicated sampling adequacy (KMO = .72). Confirmatory factor analyses (CFA) were modeled as six factors, based on four items each (with one fixed parameter), without modification indices. No attempts to improve model fits were undertaken. Thereafter, to assess whether personality traits can predict work engagement, a regression model was conducted using work engagement as the dependent variable and the six personality traits and cognitive ability as predictors.

### 2.7. Ethical Consideration

The data used for this study were collected following Lund University’s internal ethical guidelines in accordance with Swedish Ethical Review Authority (Etikprövningsmyndigheten). Data collection did not involve manipulation or deception tactics, and participation was completely voluntary and anonymous.

## 3. Results

### 3.1. Descriptive Statistics and Psychometric Properties

As shown in Table 1, the univariate normality of the data was assumed, as the skewness was below the absolute value of three and the kurtosis was below the absolute value of ten (Tabachnick et al. 2007). The skewness (−1.3) and kurtosis (2.3) were, however, markedly different concerning openness in the Mensa sample compared to the Mturk sample. In addition, the reliability of the Mini-IPIP6 and UWES-9 were examined by Cronbach’s alpha values. All dimensions from the Mini IPIP6 showed good estimates for reliability, except for openness to experience, which was slightly below .70 (Nunnally and Bernstein 1994).

The results show that the Mensa sample had considerably higher cognitive ability (*d* = 1.4) than the Mturk comparison group. Similarly, the Mensa sample was found to have significantly higher openness (*d* = .5) and honesty-humility (*d* = .65). They also showed slightly less extraversion (*d* = −.2) than the Mturk sample.

**Figure 1:**
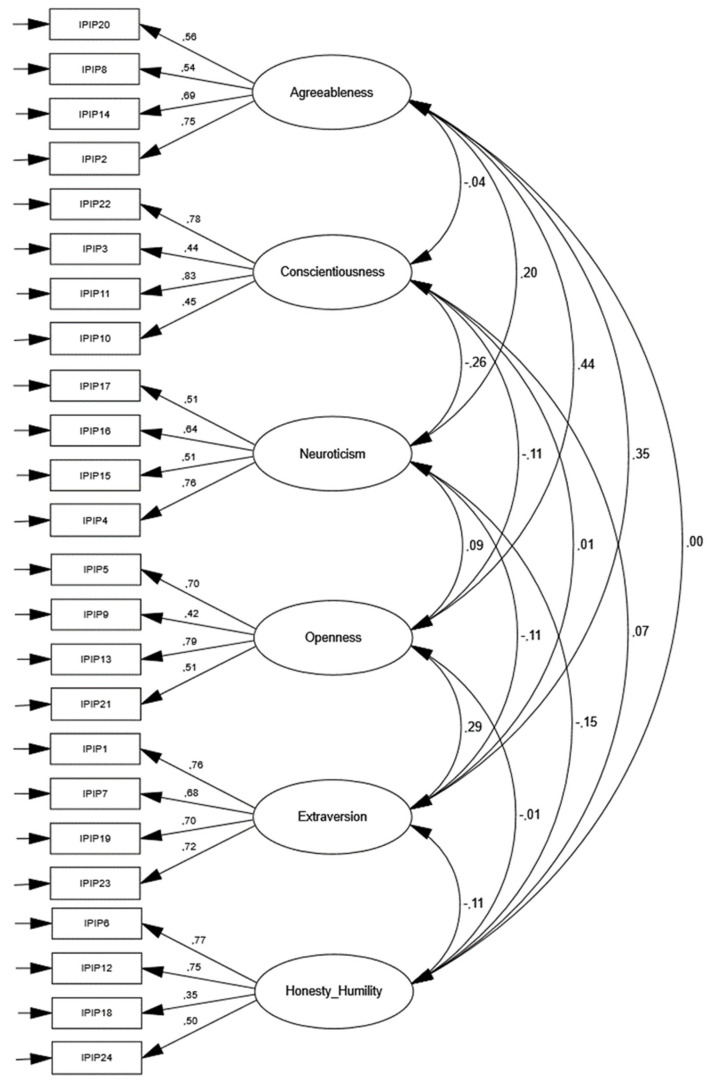
CFA of Mensa sample (N = 353). For item labels, see Sibley et al. (2011).

Conducting a confirmatory factor analysis (CFA) on the Mensa sample with all six factors based on all 24 items (χ^2^ (237) = 764.0, *p* < .001) showed a weak fit (RMSEA = .08, CFI = .78). See Figure 1 for all standardized loadings. Similarly, the control Mturk sample (χ^2^ (237) = 721.0, *p* < .001) showed fit indices of RMSEA = .08 and CFI = .78. These are expected model fits in personality inventories (Hopwood and Donnellan 2010). No modification indices were used. Overall, the items loaded normally (λ = .63) on respective trait factors, with a few exceptions in the honesty-humility items. All factor loadings can be further inspected in Appendix A Table A1 and Table A2. 

### 3.2. Personality Traits and Work Engagement

The second aim was to assess the relationship between personality traits and work engagement, comparing the Mensa sample to the control Mturk sample. A correlation table showing both samples is presented in Table 2. The relationship between cognitive ability (ICAR) and openness was higher in the Mturk sample (*r* = .33 compared to *r* = .09). Among the personality traits in the Mini-IPIP6, honesty-humility and openness stood out, reporting significantly different relationships with work engagement between samples. Mensa membership, as a moderator, showed a significant interaction effect with openness on work engagement (UWES) (β = −.29, SE = .10, Z = −2.9, *p* = .003). A simple slope analysis showed that openness was significant only in the control group. Similarly, in the control group, honesty-humility showed a significant interaction effect on work engagement (β = .19, SE = .08, Z = 2.4, *p* = .015). 

In conclusion, two multiple regression analyses, one for each sample, were conducted to examine whether personality (controlling for all traits and cognitive ability) could predict work engagement. The model predicted work engagement with statistical significance in both samples. The personality traits in the Mensa sample (F (8, 344) = 6.80, *p* < .001, R^2^ = .14) explained less variance than in the Mturk sample (F (8, 275) = 9.61, *p* < .01, R^2^ = .22). All VIFs were below 1.30. Work engagement was positively predicted by agreeableness, conscientiousness, and extraversion in both samples. Openness was a unique and significantly different predictor in the Mturk sample (β = .25), and honesty-humility showed significantly different directions of effect compared to the Mensa sample. See Table 3 for the beta weights (β) and significance testing (Z) between the Mturk and the Mensa samples.

## 4. Discussion

This study aimed to explore the psychometric properties and predictive relationships between personality traits and work engagement in gifted individuals. The Mini-IPIP6 scale proved to be a reliable and suitable instrument for assessing Mensa members’ personality traits compared to a control Mturk sample. The reliabilities were sufficient, especially given that short, four-item scales were used for each personality trait. Arguably, this is also suggested by the CFI model fit guidelines (Fabrigar et al. 1999; Hu and Bentler 1999). It is rare that personality inventories demonstrate solid model fits, partly due to the self-describing and covarying nature of items (Hopwood and Donnellan 2010). Comparing the structural validity in the present Mensa sample with other samples such as Dinić (2018) or Sibley et al. (2011) shows that the model fit RMSEA values were similar across studies (RMSEA = .06–.08). However, Dinić (2018) also reported CFI = .92, which was notably higher than in the present Mensa sample (CFI = .78). Furthermore, the CFA loadings were mostly similar to the compared studies (.50–.70), with a few exceptions, indicating that the Mini-IPIP6 items can perform in a Mensa sample. However, interestingly, the results of the honesty-humility items differed somewhat between the Mensa and Mturk groups in the present study. Loadings from items pertaining to luxury and visibility did not contribute adequately (≤.50) to the latent factor trait in the Mensa sample, while in the control Mturk group items referring to entitlement and deservedness were lower (<.40). Speculatively, this could be due to high-IQ individuals having greater access to or feeling closer to such things, leading to less variance, while the Mturk group may have considered these items to be socially undesirable (i.e., boasting) to a greater extent.

The predictive relationship between the personality traits and work engagement was similar in both groups. Overall, the models for the respective groups were able to significantly predict work engagement with higher extraversion, conscientiousness, and agreeableness. This is much in line with the previous literature (e.g., Karimi 2019). Lower neuroticism was predictive in the Mensa group, and higher openness was predictive in the Mturk group. While humility as a personality trait in employees has yet to be explored in terms of engagement, we could not report significant findings for this trait. Interestingly, openness to experience was not found to be significantly related to work engagement in the Mensa group. One simple statistical explanation for this may be the homogeneity of the sample (all scoring high in openness), which did not leave enough variance for prediction (n.b., positive kurtosis was 2.3). All in all, with the exception of the Mensa members showing notably higher openness and honesty-humility traits, these findings suggest that gifted individuals may not differ from the average population in the way their work engagement relates to their personality after all. 

### Limitations

The higher honesty-humility in Mensa members may have been partly driven by a self-selection bias in that those who chose to take the time to finish a rather difficult cognitive test battery, such as the rotating cubes in the ICAR, scored higher on this benevolent trait. Openness, in contrast, is a well-known correlate with cognitive ability (e.g., Ziegler et al. 2012) and thus was expected to be higher in a Mensa group. However, the skewness in openness in the Mensa sample may imply a homogenous range with ceiling effects compared to the more normally distributed Mturk sample. Those choosing to participate in a study such as this may be characterized by having particularly high openness. The differences in the predictive validity of openness in the present study are most likely due to the restriction of the range. Furthermore, the type of occupation was assumed to be highly varied and, accordingly, was not analyzed. It is likely that some types of workplaces would have been more impacted by high cognitive ability, while others would not have been impacted as much. Another limitation might be the use of a short personality inventory such as the Mini-IPIP6. While it had sufficient reliabilities and factor loadings, it may not yield optimal robustness for the predictive regression models. Moreover, the scope of content in a respective trait is smaller with short scales. However, longer personality forms could have even further reduced the number of participants.

Similarly, cognitive ability was measured with eight items and not the full ICAR-16 items, which may limit comparisons to studies using the full ICAR-16. Four of these items were rotating cubes, which showed difficulty levels sufficient to differentiate both within as well as between the Mensa sample and the Mturk sample. 

## 5. Conclusions

In addressing a potential gap in intelligence research, we suggest the need for a greater understanding of how gifted people engage with their work. This study may set a precedent for using the Mensa Society as a proxy for intelligence research. The results demonstrate that the relationship between personality traits and work engagement with Mensa members is similar to that of regular people, with the exception that neither openness nor honesty-humility were related to work engagement in highly intellectual individuals. The implementation of personality research in organizational development has the potential to improve engagement in workplaces. Hopefully, this study will help grow the small body of intelligence research that studies the unique characteristics of Mensa members. We hope not only to contribute to the efforts of better understanding gifted individuals but to inspire others to research the topic as well.

## Figures and Tables

**Table 1:** Descriptive statistics of Mturk (*n* = 286) and Mensa samples (*n* = 353).

Variable	Mensa	M	SD	Skew	Kurtosis	Between-Group *t*-Test	*p*	Cohen’s d
ICAR	0	.49	.21	.21	−.31	−17.31	<.001	−1.38
	1	0.78	0.20	−.80	−.21			
Open.	0	5.46	1.05	−.62	.11	−6.16 *	<.001	−.49
	1	5.95	.92	−1.27	2.26			
Neuro.	0	3.52	1.21	.11	−.12	1.34	.18	0.11
	1	3.40	1.10	.16	−.51			
Extra.	0	3.56	1.38	.02	−.76	−2.12 *	.04	−0.17
	1	3.78	1.26	−.02	−.30			
Agree.	0	5.06	1.23	−.42	−.03	.49 *	.62	0.04
	1	5.02	1.08	−.37	−.38			
Consc.	0	4.73	1.16	−.08	−.39	1.4	.16	0.11
	1	4.60	1.20	−.26	−.43			
Hon-Hum.	0	4.35	1.32	−.08	−.49	−8.14 *	<.001	−0.65
	1	5.15	1.15	−.48	−.12			
UWES	0	3.68	1.26	−.41	−.14	−1.89 *	.06	−0.15
	1	3.87	1.21	−.60	−.47			

Note. 0 = Mturk sample, 1 = Mensa sample. * Adjusted t-values (Welch’s test) due to unequal variances based on Levene’s test. Mini-IPIP (1–7). UWES (0–6). ICAR (0–1, based on four items from ICAR-16).

**Table 2:** Correlations between study variables.

Variable	1		2		3		4		5		6		7	
	0	1	0	1	0	1	0	1	0	1	0	1	0	1
1. ICAR	*.78*	*.8*												
2. Open.	.03	.05	*.82*	*.69*										
3. Neuro.	.05	−.11	−.2	.05	*.84*	*.69*								
4. Extra.	−.10	.01	.14	.22	−.21	−.13	*.89*	*.81*						
5. Agree.	−.02	−.01	.35	−.07	−.11	−.20	.20	.01	*.90*	*.73*				
6. Consc.	−.11	.04	.20	.34	−.42	.11	.01	.33	.19	.00	*.84*	*.72*		
7. Hon−Hum.	.15	−.05	.03	.04	−.07	−.06	−.30	−.13	.19	.04	.08	.03	*.84*	*.70*
8. UWES	−.02	−.04	.33	.09	−.23	−.18	.28	.25	.16	.18	.27	.19	−.12	.08

Note. 0 = Mturk sample, 1 = Mensa sample. *r* > .10 is significant at *p* < .05, and *r* > .16 is significant at *p* < .001. Cronbach’s alphas are italicized in the diagonal. UWES α = .94 and .94. Mean inter-item correlations for Mensa sample: agree. = .40, consc. = .39, neuro. = .36, open. = .37, extra. = .51, hon-hum. = .37. Mean inter-item correlations for Mturk sample: agree. = .55, consc. = .41, neuro. = .41, open. = .36, extra. = .54, hon-hum. = .40. The 95% confidence intervals were ±.07.

**Table 3:** Significance testing of differences between the impacts of personality traits in the Mturk and Mensa samples for predicting work engagement (UWES).

Predictor	β_0_	β_1_	Z-Test	*p*
Intercept				
ICAR	.03	−.06	1.13	<.13
Open.	.25	−.01	3.31	<.001
Neuro.	−.07	−.15	1.01	<.16
Extra.	.20	.20	.00	<.50
Agree.	.10	.13	−.38	<.35
Consc.	.20	.14	.77	<.22
Hon-Hum.	−.09	.08	−2.13	<.02

Note. Β_0_ = Mturk sample, β_1_ = Mensa sample. β > .10 is significant at *p* < .05, and β > .16 is significant at *p* < .001.

## Data Availability

The data used in this study are available upon reasonable request.

## Appendix A

**Table A1:** Factor loadings of CFA (Mensa sample).

Factor	Indicator	Estimate	SE	Z	*p*	Stand. Estimate
Agree.	IPIP2	1.000 ^a^				.746
	IPIP8	.726	.1010	7.19	<.001	.538
	IPIP14	1.008	.0894	11.27	<.001	.694
	IPIP20	.831	.1180	7.05	<.001	.562
Consc.	IPIP10	1.000 ^a^				.455
	IPIP22	2.052	.2701	7.60	<.001	.783
	IPIP11	2.091	.2742	7.63	<.001	.831
	IPIP3	1.031	.1726	5.98	<.001	.438
Neuro.	IPIP17	1.000				.510
	IPIP16	1.034	.1522	6.79	<.001	.642
	IPIP15	.795	.1172	6.79	<.001	.510
	IPIP4	1.424	.2038	6.99	<.001	.760
Open.	IPIP21	1.000 ^a^				.509
	IPIP13	1.384	.1813	7.63	<.001	.787
	IPIP9	.624	.1008	6.19	<.001	.418
	IPIP5	1.725	.2331	7.40	<.001	.698
Extra.	IPIP23	1.000 ^a^				.721
	IPIP19	.882	.0910	9.69	<.001	.701
	IPIP7	.904	.0968	9.33	<.001	.675
	IPIP1	.994	.0780	12.74	<.001	.761
Hon-Hum.	IPIP24	1.000				.501
	IPIP12	1.464	.2014	7.27	<.001	.748
	IPIP18	.647	.1198	5.41	<.001	.352
	IPIP6	1.344	.1910	7.04	<.001	.773

^a^ Note. *n* = 353. χ^2^ (237) = 764.0, *p* < .001. RMSEA = .08, CFI = .78.

**Table A2:** Factor loadings of CFA (Mturk sample).

Factor	Indicator	Estimate	SE	Z	*p*	Stand. Estimate
Agree.	IPIP2	1.000 ^a^				.723
	IPIP8	1.190	.1169	10.17	<.001	.780
	IPIP14	1.047	.0960	10.91	<.001	.679
	IPIP20	1.231	.1231	10.00	<.001	.783
Consc.	IPIP10	1.000 ^a^				.507
	IPIP22	1.655	.2333	7.10	<.001	.729
	IPIP11	1.505	.2222	6.77	<.001	.709
	IPIP3	1.221	.1807	6.76	<.001	.596
Neuro.	IPIP17	1.000 ^a^				.467
	IPIP16	1.404	.2159	6.50	<.001	.687
	IPIP15	1.369	.2020	6.78	<.001	.682
	IPIP4	1.685	.2558	6.59	<.001	.760
Open.	IPIP21	1.000 ^a^				.706
	IPIP13	.750	.1376	5.45	<.001	.545
	IPIP9	.825	.0989	8.35	<.001	.640
	IPIP5	.680	.1371	4.96	<.001	.480
Extra.	IPIP23	1.000 ^a^				.711
	IPIP19	1.034	.1002	10.32	<.001	.754
	IPIP7	1.101	.1066	10.33	<.001	.753
	IPIP1	.910	.0853	10.66	<.001	.710
Hon-Hum.	IPIP24	1.000 ^a^				.865
	IPIP12	.394	.0617	6.38	<.001	.397
	IPIP18	1.054	.0785	13.42	<.001	.863
	IPIP6	.317	.0665	4.77	<.001	.312

^a^ Note. *n* = 286. χ^2^(237) = 721.0, *p* < .001. RMSEA = .08. CFI = .78.
